# Identification of Autophagy-Related Biomarkers and Diagnostic Model in Alzheimer’s Disease

**DOI:** 10.3390/genes15081027

**Published:** 2024-08-05

**Authors:** Wei Xu, Xi Su, Jing Qin, Ye Jin, Ning Zhang, Shasha Huang

**Affiliations:** School of Advanced Materials Engineering, Jiaxing Nanhu University, Jiaxing 314001, China; suxi1217@jxnhu.edu.cn (X.S.); jingqin@jxnhu.edu.cn (J.Q.); 202145895126@jxnhu.edu.cn (Y.J.); 202245895107@jxnhu.edu.cn (N.Z.); 202245895108@jxnhu.edu.cn (S.H.)

**Keywords:** Alzheimer’s disease, differentially expressed autophagy genes, PPI construction, hub genes, biomarker, diagnostic model

## Abstract

Alzheimer’s disease (AD) is the most prevalent neurodegenerative disease. Its accurate pathogenic mechanisms are incompletely clarified, and effective therapeutic treatments are still inadequate. Autophagy is closely associated with AD and plays multiple roles in eliminating harmful aggregated proteins and maintaining cell homeostasis. This study identified 1191 differentially expressed genes (DEGs) based on the GSE5281 dataset from the GEO database, intersected them with 325 autophagy-related genes from GeneCards, and screened 26 differentially expressed autophagy-related genes (DEAGs). Subsequently, GO and KEGG enrichment analysis was performed and indicated that these DEAGs were primarily involved in autophagy–lysosomal biological process. Further, eight hub genes were determined by PPI construction, and experimental validation was performed by qRT-PCR on a SH-SY5Y cell model. Finally, three hub genes (*TFEB*, *TOMM20*, *GABARAPL1*) were confirmed to have potential application for biomarkers. A multigenic prediction model with good predictability (AUC = 0.871) was constructed in GSE5281 and validated in the GSE132903 dataset. Hub gene-targeted miRNAs closely associated with AD were also retrieved through the miRDB and HDMM database, predicting potential therapeutic agents for AD. This study provides new insights into autophagy-related genes in brain tissues of AD patients and offers more candidate biomarkers for AD mechanistic research as well as clinical diagnosis.

## 1. Introduction

Alzheimer’s disease (AD) is currently the most prevalent neurodegenerative disease [1]. Various hallmarks of AD indicate its multifactorial nature, such as hyperphosphorylated tau protein, deposits of amyloid-β (Aβ) around neurons, dyshomeostasis of biometals, oxidative stress, chronic nerve inflammation, and so on [2,3]. Although a great deal of investigation has been carried out, the accurate pathogenic mechanisms of AD have not been completely clarified, and effective therapeutic treatment is still inadequate. The number of AD patients aged 65 and older is predicted to reach 7.2 million by 2025 [4].

A number of studies have found that autophagy plays an important role in neurological disorders such as Alzheimer’s. As a catabolic process that delivers and degrades intracellular b, autophagy maintains cellular homeostasis by degrading non-essential proteins and organelles and recycling components. Additionally, starvation, oxidative stress, and a variety of diseases can induce it [5,6,7,8]. There are three main types of autophagy in mammalian cells: macroautophagy, microautophagy, and chaperon-mediated autophagy. Of these, macroautophagy is the predominant and best-studied type, and is generally referred to as “autophagy” [9]. Misfolded proteins, including Aβ and Tau, are accumulated and aggregated in AD, and they are degraded by autophagy and the ubiquitinproteasome system (UPS). When the UPS is overloaded or damaged in AD, autophagy is used to clear excessive misfolded proteins [10,11].

Aβ and phosphorylated Tau appear to cause abnormal autophagy and mitophagy in AD [12]. When the hippocampus or ventral tegmental area (VTA) is aging or affected by AD, neuronal autophagy activity is decreased, resulting in Aβ accumulation. Restoring autophagy reduces Aβ levels and reverses cognitive decline and neuronal degeneration [13,14]. It has been shown that activation of autophagy in AD leads to a decrease in the accumulation of Aβ and Tau proteins in the cytoplasm [15,16]. In addition, neuroinflammation contributes significantly to AD, and anti-inflammatory therapy represents a viable treatment option [17,18]. It has been reported that inflammation mediated by microglia is thought to affect neurodegenerative diseases through autophagy [19,20,21,22]. Altogether, autophagy is closely associated with AD and plays multiple roles in eliminating harmful aggregated proteins and maintaining cell homeostasis. Exploring autophagy-related gene expression in AD may provide us with new insights into pathological mechanism, diagnosis, and treatment for this disease.

AD-related phenotypes are increasingly utilized to identify differentially expressed genes (DEGs) using bioinformatics methods. Phenotype-associated DEGs in AD have provided many potential biomarkers for mechanistic research and clinical diagnosis. Zhao et al. [23] identified 18 ferroptosis-related hub genes in AD and explored their potential as diagnostic markers. Yan et al. [24] screened two mitochondrial-related candidate genes as diagnostic markers for late-onset Alzheimer’s disease (LOAD) as well as mild cognitive impairment (MCI), and constructed a LOAD diagnostic prediction model. Zhang et al. [25] identified five hub genes related to the oxidative stress (OS) process in AD, constructed a diagnostic model, and predicted hub gene-targeted drugs as well as miRNAs as potential treatments. Du et al. [26] identified five blood biomarkers and constructed a copper metabolism-associated polygenic prediction model. Gu et al. [27] screened nine genes linking AD and iron metabolism from brain issues, and constructed a multigenic prediction model that was further validated in blood samples. Qin et al. [28] identified nine differentially expressed autophagy-related genes (DEAGs) in peripheral blood based on GSE63060 and GSE63061 datasets, and developed a personalized nomogram model by combining with age and sex. Li et al. [29] found 10 DEAGs based on GSE63061 and GSE140831 datasets, and evaluated their potentiality for AD biomarkers. However, the two investigations of DEAGs were both based on blood samples, and how autophagy-related genes varies in brain issues is worth exploring.

**Figure 1:**
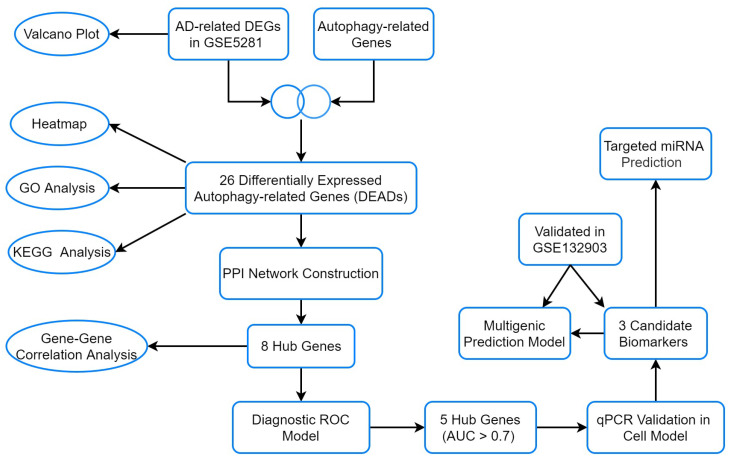
The flow chart of the analyses.

This study investigated differentially expressed autophagy-related genes (DEAGs) from brain issues based on GEO database GSE5281, explored their biological function, detected hub genes using bioinformatics methods, and validated them by qRT-PCR on a cell model. Finally, a diagnostic model was established and validated in an external dataset (Figure 1). Our findings provide new insights into autophagy-related genes in brain tissues of AD patients and offer more candidate biomarkers for AD mechanistic research as well as clinical diagnosis.

## 2. Materials and Methods

### 2.1. Data Acquisition

AD-associated microarray datasets were obtained from the GEO database (http://www.ncbi.nlm.nih.gov/geo/, accessed on 11 February 2024). The GSE5281 gene expression profile contained 87 AD samples and 74 healthy controls. The GSE132903 gene expression profile was used for validation, which included 97 AD samples and 98 healthy controls. All samples in the two datasets were extracted from brain tissue. Autophagy-related gene sets were downloaded from the GeneCards (https://www.genecards.org/, accessed on 11 February 2024) database. Genes with a relevance score > 4 were selected as the highly associated genes for autophagy to facilitate subsequent difference analysis.

### 2.2. Identification of DEGs and DEAGs

The Limma package in R (4.2.1) was applied for standardization and analysis of DEGs between AD samples and control subjects [30]. The screening condition was predetermined as logFC values > 1 and adjusted *p*-values < 0.05. Volcano plots were performed to visualize the expression of DEGs using the ggplot2 (3.3.6) package in R (4.2.1) software. Additionally, a Venn diagram and heatmap were created to describe the DEAGs, which were obtained from the intersection of DEGs in GSE5281 and 325 autophagy-related genes.

### 2.3. Biological Functional and Enrichment Analysis of DEAGs

To clarify the potential biological processes and molecular functions of DEAGs, Gene Ontology (GO) and Kyoto Encyclopedia of Genes and Genomes (KEGG) pathway enrichment were performed using the R clusterProfiler (4.4.4) package [31], and the results were visualized using the ggplot2 (3.3.6) package in R (4.2.1) software.

### 2.4. PPI Network Construction and Hub Gene Identification

A protein–protein interaction (PPI) network was constructed to predict the interaction among the DEAGs using the STRING database (https://string-db.org/, accessed on 11 February 2024) [32]. Cytoscape software (Version 3.10.2) was involved to detect PPI pairs (confidence score > 0.4) and visualize the results. The hub genes were screened by a MCODE analysis module using the default parameters (degree cutoff = 2, node score cutoff = 0.2, K-core = 2, max. depth = 100). The interaction relationships among hub genes were analyzed based on Pearson correlation statistics, and the results were drawn as a heatmap using the ggplot2 (3.3.6) package in R (4.2.1) software.

### 2.5. Diagnostic ROC Curve Construction

Logistic regression was performed to evaluate the diagnostic significance of the hub genes. A response variable was assigned 1 for AD samples and 0 for ND (non-demented) controls. Receiver operating characteristic (ROC) analysis was performed using the pROC (1.18.0) package in R (4.2.1) software, and the results were visualized by ggplot2 [3.3.6]. The diagnostic efficacy was assessed by the area under the curve (AUC).

### 2.6. Cell Culture and qRT-PCR

The SH-SY5Y neuronal cell line was used as the validated cell model by quantitative real-time PCR (qRT-PCR) analysis. SH-SY5Y cells were cultured in DMEM supplemented with 10% heat-inactivated fetal calf serum, 100 IU/mL penicillin, and 100 μg/mL streptomycin at 37 °C in humidified 5% CO_2_ air. Then, Aβ1–42 with a final concentration of 8 μM were added to induce SH-SY5Y cells for 12 h. Trizol Reagent (Takara, Japan) was used to extract total RNA, and then cDNA was reversed-transcribed with PrimeScript RT Master Mix (Takara, Japan) according to the manufacturer’s instructions.

Real-time PCR was performed with the following procedure: denaturation at 95 °C for 30 s followed by 40 cycles of denaturation (95 °C, 5 s), annealing (55 °C, 30 s), and extension (72 °C, 30 s). Relative expression changes were calculated using the 2^−∆∆CT^ formula, and GAPDH was used as the internal control for normalization. Statistical analysis was performed using the Welch *t*-test on GraphPad Prism 8.0.0. The significance levels were given as follows: *** *p* < 0.001; ** *p* < 0.01; * *p* < 0.05. 

### 2.7. External Dataset Validation

The differential expression of qRT-PCR-validated hub genes was further verified in external dataset GSE132903. Statistical analyses were performed using the Mann–Whitney U test, and the results was visualized as violin plots using ggplot2 (3.3.6), stats (4.2.1), and the car (3.1-0) R package in R (4.2.1) software.

### 2.8. Exploration of microRNAs Targeting the Hub Genes

Potential miRNAs targeting the qRT-PCR-validated hub genes were obtained by the miRDB database (https://mirdb.org/cgi-bin/search.cgi/, accessed on 11 February 2024). The target score was set above 85 to screen the miRNAs of higher relevance. The Human microRNA Disease Database (HMDD) [33] was used to investigate and validate the association between these screened miRNAs and AD.

## 3. Results

### 3.1. Identification of DEAGs

**Figure 2:**
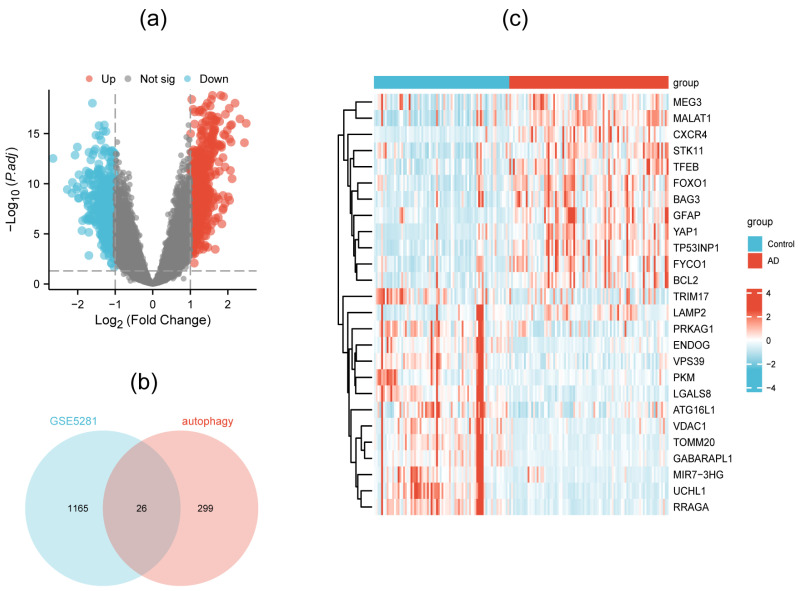
Volcano plot showing differential gene analysis in the GSE5281 dataset (**a**), Venn diagram indicating 26 DEAGs (**b**), and heatmap exhibiting the expression levels of DEAGs in AD and normal samples (**c**).

Through differential gene analysis performed in the GSE5281 dataset, 1191 DEGs were distinguished, which are shown in a volcano plot (Figure 2a). When the relevance score was above 4, 325 autophagy-related genes were screened out from the autophagy database. Finally, 26 DEAGs were obtained when comparing 1191 DEGs and 325 ATGs, as exhibited in the Venn diagram (Figure 2b). The expression levels of the 26 DEAGs presented an obvious difference between the AD patients and the normal persons (Figure 2c, Table S1).

### 3.2. GO and KEGG Enrichment Analysis of DEAGs

**Figure 3:**
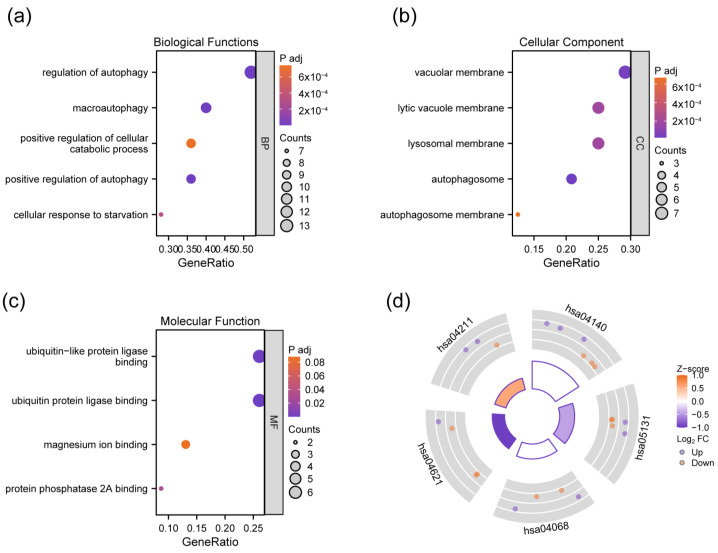
Bubble plot of GO analyses showing biological process (**a**), the cellular component (**b**), and the molecular function (**c**) of DEAGs, and circle plot of KEGG analysis indicating involved pathways of DEAGs (**d**). hsa04140: Autophagy—animal; hsa05131: Shigellosis; hsa04068; FoxO signaling pathway; hsa04621: NOD-like receptor signaling pathway; hsa04211: longevity-regulating pathway.

To clarify the potential biological functions of DEAGs, enrichment analyses were performed. According to the results, DEAGs were mainly involved in the biological process (BP) of “regulation of autophagy”, “positive regulation of autophagy”, “macroautophagy”, “cellular response to starvation”, and “positive regulation of cellular catabolic process” (Figure 3a). Cellular component (CC) enrichment revealed that DEAGs played a role in the vacuolar membrane, lysosomal membrane, lytic vacuole membrane, and autophagosome membrane (Figure 3b). Molecular function (MF) mainly comprises ubiquitin protein ligase binding, ubiquitin-like protein ligase binding, protein phosphatase 2A binding, and magnesium ion binding (Figure 3c). KEGG analysis indicated the involved pathways, including autophagy (autophagy—animal), shigellosis, FoxO signaling pathway, NOD-like receptor signaling pathway, and longevity-regulating pathway (Figure 3d). The overall results of GO and KEGG enrichment analysis are shown in Table S2.

### 3.3. PPI Network Construction and Hub DEAG Detection

**Figure 4:**
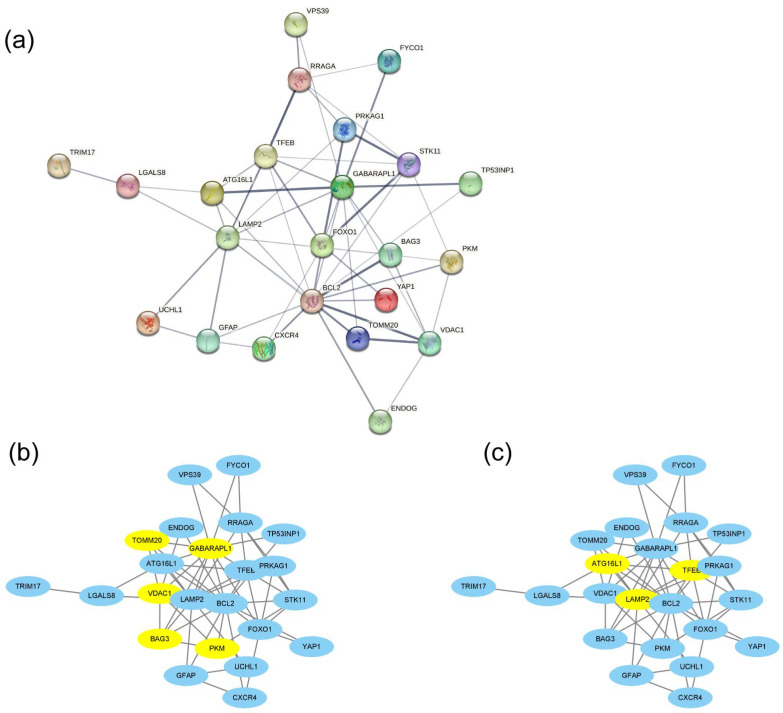
PPI network analysis of 26 DEAGs constructed using STRING (**a**) and 8 hub genes in two cluster networks determined using the MCODE analysis module of Cytoscape (**b**,**c**). Hub genes are highlighted in yellow.

**Figure 5:**
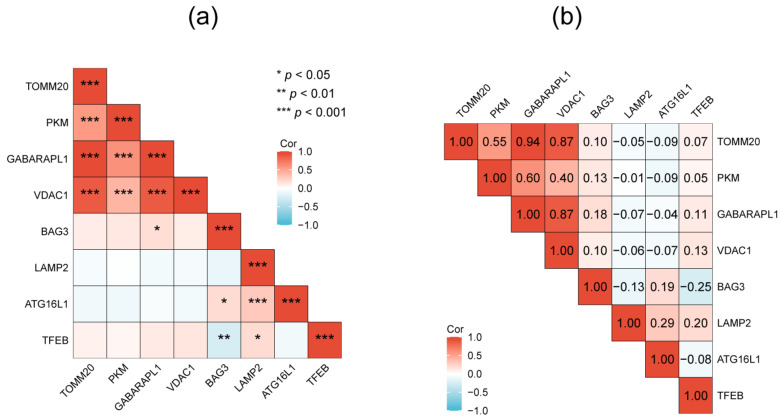
The relationships among the 8 hub genes evaluated by *p*-value (**a**) and coefficient of correlation (**b**).

The PPI network of the 26 DEAGs was established using STRING (Figure 4a). The top eight highest-scored genes were selected as the hub genes in two cluster networks using the MCODE analysis module of Cytoscape, such as *BAG3*, *GABARAPL1*, *PKM*, *TOMM20*, and *VDAC1* in MCODE-1, as well as *ATG16L1*, *LAMP2*, and *TFEB* in MCODE-2 (Figure 4b,c; Table 1). The relationships among the eight hub genes are shown in Figure 5 and Tables S3 and S4. *TOMM20* showed the highest positive correlation with *GABARAPL1* (PCC = 0.94), while *BAG3* exhibited the highest negative correlation with *TFEB* (PCC = −0.25).

### 3.4. Diagnostic ROC Model for Hub Genes

**Figure 6:**
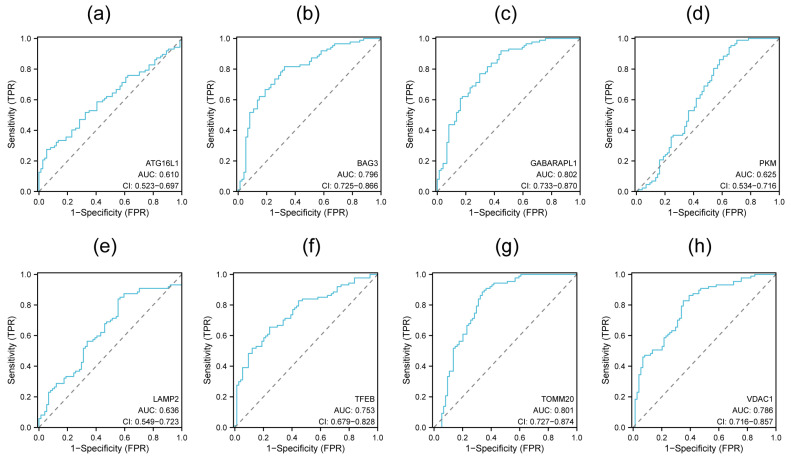
ROC curves for each hub gene. (**a**) *ATG16L1*, (**b**) *BAG3*, (**c**) *GABARAPL1*, (**d**) *PKM*, (**e**) *LAMP2*, (**f**) *TFEB*, (**g**) *TOMM20*, (**h**) *VDAC1*.

The ROC curve was used to evaluate the potential diagnostic application of each hub gene. The AUC values range from 0.5 to 1, and being closer to 1 suggests more accuracy. Models with a value of 0.7 were considered reasonable and those with values > 0.8 were considered strong. The results indicate that the AUC values of all hub genes were above 0.6. The values of *TFEB*, *BAG3*, and *VDAC1* were above 0.7, and those of *TOMM20* and *GABARAPL1* were above 0.8 (Figure 6). The ROC curves suggest a potential diagnostic value for these five hub genes.

### 3.5. Validation of Hub Genes by qRT-PCR and External Dataset

**Figure 7:**
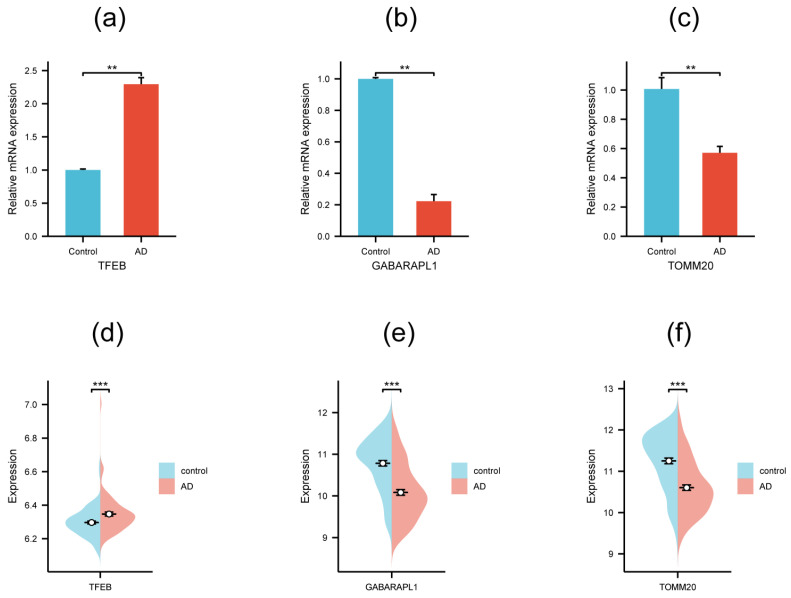
qRT-PCR validation (**a**–**c**) and external dataset validation in GSE132903 (**d**–**f**) of the three hub genes. Significance levels were given as follows: *** *p* < 0.001; ** *p* <0.01.

To further confirm the reliability of the prediction results, five hub genes with AUC > 0.7 were selected to be validated on the SH-SY5Y cell model. qRT-PCR was performed to monitor their mRNA expression level (Table 2), and the results demonstrated that three genes of the five showed a significant differential expression level between the AD cell model and the control group (Figure 7a–c). *TFEB* exhibited an obviously higher mRNA expression level in the AD cell model compared with the control group, while *TOMM20* and *GABARAPL1* showed a distinctly lower mRNA expression level. Next, GSE132903 was involved as the external verifying dataset to further validate the three genes above. The analysis indicated that all of them displayed a significant expression difference between the AD patient and control groups (Figure 7d–f). The changing trends of *TFEB*, *TOMM20*, and *GABARAPL1* in the qRT-PCR experiment and the external dataset were all consistent with the tendency in the GSE5281 dataset (Table 1), suggesting the three genes possessed potential values to be candidate biomarkers for AD mechanism research and clinical diagnosis.

### 3.6. Multigenic Prediction Model Construction and Validation

**Figure 8:**
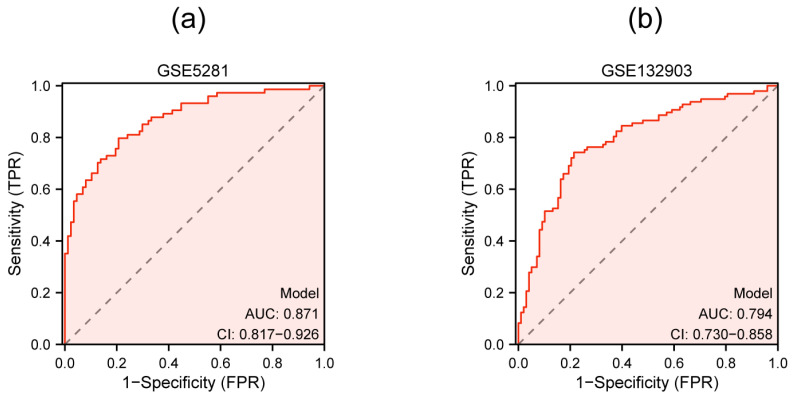
Multigenic prediction model constructed by three qRT-PCR-validated genes in the GSE5281 dataset (**a**) and validated in the GSE132903 dataset (**b**).

A multigenic prediction model was constructed based on *TFEB*, *TOMM20*, and *GABARAPL1* in the GSE5281 dataset. The results show that the AUC value of the ROC curves was 0.871, demonstrating the good predictive ability of the model (Figure 8a). Next, we further validated this model in the GSE132903 dataset and the AUC was 0.794, which confirmed the predictive accuracy of this diagnostic model (Figure 8b).

### 3.7. Identification of miRNAs Targeting Autophagy-Related Biomarkers

We utilized the miRDB database to investigate the potential miRNAs targeting *TFEB*, *TOMM20*, and *GABARAPL1*. When the target score was set above 85, a total of 54 miRNAs were screened out. Then, the HMDD database was involved to further validate the association between these detected miRNAs and AD. The results show that 13 miRNAs were closely relevant to AD (Table 3), which suggests that they may hold promise as potential therapeutic agents for this disease.

## 4. Discussion

Alzheimer’s disease is a progressive brain disease and the most common cause of dementia. The accumulation of Aβ outside neurons as well as tau of unusual form inside neurons are two main brain changes in AD [4]. The autophagy process plays a key role in cell and tissue homeostasis, as well as in aging and many diseases, including Alzheimer’s and neurodegenerative diseases [47,48]. In mild autophagy, damaged organelles and aggregates of proteins are removed from the cell, thus limiting the spread of toxins [19]. Therefore, exploring differentially expressed autophagy-related genes (DEAGs) in AD by informatics methods would facilitate the understanding of the mechanism and finding novel biomarkers for this disease. 

In this study, we identified 1191 DEGs based on brain issues of 87 AD and 74 healthy controls obtained from GSE5281 dataset. Then, these DEGs were intersected with 325 autophagy-related genes from GeneCards and 26 DEAGs were screened, including 13 upregulated genes and 13 downregulated ones. Subsequently, GO and KEGG enrichment analysis was performed, and the results indicate that DEAGs were primarily involved in the autophagy–lysosomal biological process, which confirmed a close relationship between autophagy and AD. Further, eight hub genes were detected by PPI construction, and their diagnostic value was evaluated by ROC curve. Experimental validation was performed by qRT-PCR to further confirm the differential expression of five genes with AUC > 0.7. Finally, three genes (*TFEB*, *TOMM20*, *GABARAPL1*) were determined to be potential candidate AD biomarkers. 

PPI network construction of DEAGs detected eight hub genes that formed two cluster networks. One included *TFEB*, *LAMP2*, and *ATG16L1*. TFEB (transcription factor EB) is a key transcriptional regulator of autophagy and lysosomal biogenesis [49]. A dysfunctional autophagy–lysosomal pathway contributes to AD progression in both patients and animal models [15,50]. LAMP2 (lysosomal associated membrane protein 2) is an important component of the lysosomal membrane [51]. Activation of its isoform LAMP2A ameliorates proteotoxicity-driven neurodegeneration and improves neuronal function when it is expressed on lysosomes [52]. The ATG (autophagy related) protein plays an essential role in proper recruitment of lysosomes and the prevention of aberrant degradation of cellular contents. ATG16L1 is a key player at various stages of autophagy due to its interaction with proteins and lipids [53]. 

The other cluster network contained five hub genes. BAG3 is a multifunctional protein involved in a range of cellular processes, such as apoptosis, development, cytoskeleton arrangement, and selective macroautophagy [54]. The selective macroautophagy pathway facilitated by BAG3 plays a crucial role in maintaining cellular protein quality by breaking down potentially harmful aggregating proteins [55,56]. GABARAP subfamily proteins (GABARAPs) belong to mammalian autophagy-related protein Atg8. GABARAPL1 is a primary mediator for selective autophagy, including glycophagy and mitophagy [57,58]. The dysfunction of mitochondria can be a sign of oxidative stress, inflammation, aging, and chronic degenerative diseases [59]. VDAC1 (voltage-dependent anion-selective channel protein 1) is an important regulator of mitochondrial function. It regulates the transport of proteins and metabolites, and coordinates apoptosis as well as other cellular stress-related processes [60]. TOMM is the translocase of the outer mitochondrial membrane, which mediates the entry of most mitochondrial proteins into the mitochondrial interior [61]. TOMM20 is an important receptor subunit of the TOMM complex and serves by recognizing mitochondrial precursor proteins with cleavable N-terminal presequences [62]. PKM (pyruvate kinase M1/2) is the main catalytic enzyme in the rate-limiting step in glycolysis for energy production [63], and PKM2 has been shown to be involved in the regulation of cognitive dysfunction via related signaling pathways [64]. 

Many miRNAs have been reported to be closely associated with AD. In this study, we also explored AD-related miRNAs targeting the three experimental validated biomarkers (*TFEB*, *TOMM20*, and *GABARAPL1*) by the miRDB database and the HMDD database. Of the miRNAs we retrieved, hsa-miR-29a-3p [34], hsa-miR-29b-3p [36], hsa-miR-143-3p [45], and hsa-miR-133b [46] were reported to be promising biomarkers for AD, which were validated either by cell model or clinical plasma samples. Some other miRNAs were found to be key factors involved in many pathological processes of AD. For example, hsa-miR-195-5p [40] and hsa-miR-155-5p [42] were implicated to play roles in regulating inflammatory responses in AD. hsa-miR-497-5p [43] was relevant to alleviating BBB permeability in AD microenvironment. hsa-miR-29c-3p [35] participated in inhibiting BACE1 expression and activating the Wnt/β-catenin pathway. Since these miRNAs were shown to target *TFEB*, *TOMM20*, or *GABARAPL1*, their underlying regulatory mechanism could be multiple and complicated. It is worth further exploring their potential to be therapeutic targets for AD treatment.

As integrating multiple biomarkers can provide comprehensive information to improve the diagnostic accuracy and specificity of AD [65], a multigenic prediction model was further established based on the three key genes. The results demonstrated good predictability (AUC = 0.871) and were verified in the GSE132903 dataset (AUC = 0.794). In the preclinical phase, although individuals have not yet developed symptoms such as memory loss, they may have measurable brain changes, which indicate the earliest signs of AD (biomarkers) [4]. Our study provides more potential biomarkers for early diagnosis of AD, which are also supplements for previous studies of DEAGs. In future, more experimental research could be performed to further explore their application, such as Western blot and immunochemistry on animal model and clinical samples. Since blood samples have easy accessibility and broad application prospects [66,67], the biomarkers detected in our study could be further verified in the clinical plasma of AD patient in future investigations.

## 5. Conclusions

In this study, a bioinformatics approach was used to identify and evaluate potential biomarkers related to autophagy in AD. Enrichment analysis indicated 26 DEAGs mainly focused on the autophagy–lysosomal biological process. PPI analysis detected eight hub genes, and ROC curves indicated that five of them had better diagnostic accuracy (AUC > 0.7). Molecular validation of qRT-PCR suggested that three hub genes (*TFEB*, *TOMM20*, *GABARAPL1*) exhibited a significant differential expression in cell model and could be potential candidate AD biomarkers. Finally, the potential miRNAs targeting these three gene were investigated, and 13 miRNAs were found to be closely relevant to AD.

## Figures and Tables

**Table 1:** Eight hub genes selected from DEAGs.

Gene Symbol	logFC	p.adj	Description
*TFEB*	1.80	1.75 × 10^−14^	Transcription factor EB
*BAG3*	1.39	2.25 × 10^−11^	BAG cochaperone 3
*LAMP2*	1.24	5.57 × 10^−13^	Lysosomal-associated membrane protein 2
*VDAC1*	−1.26	3.96 × 10^−7^	Voltage-dependent anion channel 1
*PKM*	−1.06	3.05 × 10^−5^	Pyruvate kinase M1/2
*ATG16L1*	−1.02	1.60 × 10^−4^	Autophagy-related 16 like 1
*TOMM20*	−1.01	1.77 × 10^−6^	Translocase of outer mitochondrial membrane 20
*GABARAPL1*	−1.01	1.57 × 10^−9^	GABA type A receptor-associated protein-like 1

**Table 2:** Primer sequences of mRNA for qRT-PCR.

Gene Symbol	Primer Sequence	
	Forward	Reverse
*TFEB*	5′-ACCTGTCCGAGACCTATGGG-3′	5′-CGTCCAGACGCATAATGTTGTC-3′
*TOMM20*	5′-GGTACTGCATCTACTTCGACCG-3′	5′-TGGTCTACGCCCTTCTCATATTC-3′
*GABARAPL1*	5′-ATGAAGTTCCAGTACAAGGAGGA-3′	5′-GCTTTTGGAGCCTTCTCTACAAT-3′
*GAPDH*	5′-CCAGCCCAGCAAGGATACTG-3′	5′-GGTATTCGAGAGAAGGGAGGGC-3′

**Table 3:** Hub gene-targeted miRNAs and the associations between AD.

Gene Symbol	miRNA Name	Description
*TFEB*	hsa-miR-29a-3p	Slightly dysregulated in plasma; potential biomarkers of AD [34].
*TFEB*	hsa-miR-124-3p	Colocalization with microglia in AD patient hippocampi; reshapes microglia plasticity; relevant with inflammation in AD-associated neurodegeneration [35].
*TFEB*	hsa-miR-29b-3p	Has diagnostic potential as minimally invasive AD biomarker [36].
*TFEB*	hsa-miR-29c-3p	Inhibits BACE1 expression; activates the Wnt/β-catenin pathway; plays a therapeutic role in AD [37].
*TOMM20*	hsa-let-7a-2-3p	Related to cognitive impairment [38].
*TOMM20*	hsa-miR-204-3p	Controls the timing of the dopaminergic differentiation [39].
*GABARAPL1*	hsa-miR-195-5p	Upregulated in the AD process [40].
*GABARAPL1*	hsa-miR-16-5p	Consistently downregulated in late-stage AD by meta-analysis across the literature [41].
*GABARAPL1*	hsa-miR-155-5p	Regulate inflammatory responses in the pathogenesis of AD [42].
*GABARAPL1*	hsa-miR-497-5p	The MEM/LINC00094/miR-224-5p (miR-497-5p)/endophilin-1 axis exerts a key role in regulating the BBB permeability in the AD microenvironment [43].
*GABARAPL1*	hsa-miR-15b-5p	Involved in platelet reactivity in AD [44].
*GABARAPL1*	hsa-miR-143-3p	Upregulated in the serum of AD patients; miR-143-3p inhibition promotes neuronal survival; the miR-143-3p/NRG1 axis is a potential therapeutic target and candidate biomarker for AD [45].
*GABARAPL1*	hsa-miR-133b	A novel promising diagnostic biomarker for AD; may have a neuroprotective role in AD and targets EGFR [46].

## Data Availability

The original contributions presented in the study are included in the article/Supplementary Materials; further inquiries can be directed to the corresponding author.

## Supplementary Materials

The following supporting information can be downloaded at: https://www.mdpi.com/article/10.3390/genes15081027/s1, Table S1: Information of 26 DEADs; Table S2: Results of GO and KEGG enrichment analysis; Table S3: Correlation coefficient of 8 hub genes; Table S4: *p*-value of Pearson correlation statistics of 8 hub genes.
