# Supplementary material for: Identification of Autophagy-Related Biomarkers and Diagnostic Model in Alzheimer’s Disease
Source: Genes (Basel). 2024 Aug 5;15(8):1027. doi: 10.3390/genes15081027 (PMC11354206; doi:10.3390/genes15081027)
Supplement: Supplementary file 1 [file genes-15-01027-s001.zip › Table S2.pdf]

Table S2 Results of GO and KEGG enrichment analysis

Ontology	ID	Description	GeneRatio	BgRatio	p.adjust
BP	GO:0010506	regulation of autophagy	13/25	336/18800	7.05×10^{-14}
BP	GO:0010508	positive regulation of autophagy	9/25	136/18800	4.2×10^{-11}
BP	GO:0016236	macroautophagy	10/25	306/18800	1.08×10^{-9}
BP	GO:0009267	cellular response to starvation	7/25	160/18800	3.27×10^{-7}
BP	GO:0031331	positive regulation of cellular catabolic	9/25	449/18800	7.42×10^{-7}
CC	GO:0005776	autophagosome	5/24	112/19594	2.24×10^{-5}
CC	GO:0005774	vacuolar membrane	7/24	449/19594	4.03×10^{-5}
CC	GO:0005765	lysosomal membrane	6/24	404/19594	0.0002
CC	GO:0098852	lytic vacuole membrane	6/24	404/19594	0.0002
CC	GO:0000421	autophagosome membrane	3/24	51/19594	0.0007
MF	GO:0031625	ubiquitin protein ligase binding	6/23	298/18410	0.0001
MF	GO:0044389	ubiquitin-like protein ligase binding	6/23	317/18410	0.0001
MF	GO:0051721	protein phosphatase 2A binding	2/23	30/18410	0.0290
MF	GO:0000287	magnesium ion binding	3/23	222/18410	0.0878
KEGG	hsa04140	Autophagy - animal	6/18	141/8164	3.73×10^{-5}
KEGG	hsa05131	Shigellosis	5/18	247/8164	0.0054
KEGG	hsa04068	FoxO signaling pathway	4/18	131/8164	0.0054
KEGG	hsa04621	NOD-like receptor signaling pathway	4/18	184/8164	0.0149
KEGG	hsa04211	Longevity regulating pathway	3/18	89/8164	0.0182
